# Nitric oxide-associated chondrocyte apoptosis in trauma patients after high-energy lower extremity intra-articular fractures

**DOI:** 10.1007/s10195-015-0350-2

**Published:** 2015-05-10

**Authors:** Daniel E. Prince, Justin K. Greisberg

**Affiliations:** Memorial Sloan Kettering Cancer Center, 1275 York Avenue, Howard 1013, New York, NY 10065 USA; New York Presbyterian Hospital, Columbia University, New York, NY USA

**Keywords:** Apoptosis, Chondrocyte, Nitric oxide, Intra-articular fractures, Post-traumatic osteoarthritis

## Abstract

**Background:**

The primary goal of this study was to identify nitric oxide (NO)-induced apoptosis in traumatized chondrocytes in intra-articular lower extremity fractures and the secondary goal was to identify the timeline of NO-induced apoptosis after injury.

**Materials and methods:**

This is a prospective collection of samples of human cartilage harvested at the time of surgery to measure apoptotic cell death and the presence of NO by immunohistochemistry. Three patients met the criteria for control subjects and eight patients sustained high-energy intra-articular fractures and were included in the study. Subjects who sustained intra-articular acetabular, tibial, calcaneal and talus fracture had articular cartilage harvested at the time of surgical intervention. All 8 patients underwent open reduction and internal fixation of the displaced intra-articular fractures. The main outcome measures were rate of apoptosis, degree of NO-induced apoptosis in chondrocytes, and the timeline of NO-induced apoptosis after high-energy trauma.

**Results:**

The percentage of apoptotic chondrocytes was higher in impacted samples than in normal cartilage (56 vs 4 %), confirming the presence of apoptosis after intra-articular fracture. The percentage of cells with NO was greater in apoptotic cells than in normal cells (59 vs 20 %), implicating NO-induction of apoptosis. The correlation between chondrocyte apoptosis and increasing time from injury was found to be −0.615, indicating a decreasing rate of apoptosis post injury.

**Conclusions:**

The data showed the involvement of NO-induced apoptosis of chondrocytes after high-energy trauma, which decreased with time from injury.

## Introduction

Post-traumatic arthritis remains a problem after intra-articular fractures. Anatomic reduction of displaced articular fragments is the gold standard to restore articular congruency. However, anatomic reduction does not guarantee viable chondrocytes and functional articular cartilage in the zone of injury. Post-traumatic arthritis develops in many patients with pilon, calcaneus, or acetabular fractures despite a good reduction [1–3]. Earlier studies of blunt trauma in canine articular cartilage found biochemical aberrations in addition to structural full-thickness damage to the cartilaginous matrix of chondrocytes when subjected to intra-articular fractures without obvious displacement of the subchondral bone [4–6]. In recent years, attention has been directed to the chondrocyte and to finding alterations in chondrocyte viability in human and animal cartilage after blunt trauma [7–9]. Chondrocytes may die by necrosis (physical disruption of the cell) or by apoptosis (programed cell death). Apoptosis was first identified in thymocytes 30 years ago [10] as a logical means of eliminating unwanted cells. Chondrocyte apoptosis is a normal physiological event in the physis, which allows osteoblasts to lay down osteoid; however, chondrocyte apoptosis has also been identified as a pathologic process in osteoarthritic articular cartilage [11–16]. It is speculated that loss of chondrocytes may be an essential step in arthritis [12], i.e., without chondrocytes to maintain the matrix, the joint inevitably progresses to end-stage arthritis. Nitric oxide (NO) can induce chondrocyte apoptosis in vitro [14, 17–20] and has been found in both rheumatoid and osteoarthritic cartilage [12]. Researchers have suggested that NO may be a signaling molecule for chondrocyte apoptosis in vivo [21].

Therefore, we hypothesized that (1) the percentage of cells with NO is greater in apoptotic chondrocytes than in non-apoptotic cells, (2) the percentage of chondrocytes with NO-induced apoptosis is greater in cartilage specimens from intra-articular fractures than in control specimens, and (3) the duration of cell death secondary to apoptosis will be determined over time.

## Materials and methods

The study design was a prospectively collected series of cartilage specimens from both high-energy intra-articular trauma patients and a control group of specimens from patients undergoing elective first tarsometatarsal fusions. The specimens were stained for viability, presence of NO, and apoptosis and the percentage of stained cells for each variable was calculated for each specimen.

The study included adult patients presenting at our institution who had sustained intra-articular (OTA type B and C) high-energy lower extremity trauma requiring open reduction and internal fixation. The cartilage specimens were from the injured area and were sufficiently small so that reconstruction of the fragment was not possible at the time of definitive surgical repair. Exclusion criteria for both control and cohort groups were age >65 years, history of prior pain, surgery or arthritis in the affected joint, diabetes or a history of systemic inflammatory disease. All control specimens were obtained from healthy young adults who met the same exclusion criteria undergoing first tarsometatarsal arthrodesis for distal hallux deformity via Lapidus fusion. One trauma patient had single medication-controlled hypertension and had previously undergone an uneventful cholecystectomy many years before. Another patient had medication-controlled gastro-esophageal reflux disease, and another patient had an anxiety disorder, while the remaining trauma and control patients had no prior preoperative medical or surgical conditions.

Eight specimens were harvested from eight patients. Four patients sustained tibial plateau fractures after being struck by a car; one patient had a talus fracture, one had a calcaneus fracture, and one had tibial pilon injury (all resulting from a fall from a height); and another had an acetabular fracture after a car accident. Specimens were immediately fixed in formalin, embedded in paraffin, and then cut into 5-μm sections in a direction that captured all levels (surface to subchondral bone). A power analysis was performed after refining the staining technique utilizing three control and three fracture specimens. The analysis determined that a total of 8 fracture specimens would be necessary for a power of 0.91. This study was approved by the Institutional Review Board and was performed in accordance with the ethical standards of the 1964 Declaration of Helsinki as revised in 2000.

Paraffin-embedded tissues were rehydrated and then permeabilized in 0.1 % triton X-100 in 0.1 % sodium citrate solution. Slides were incubated with 0.5 % sheep testicular hyaluronidase (Sigma-Aldrich, St Louis, MO, USA). Staining was performed with a dUTP terminal transferase-mediated nick-end labeling (TUNEL) assay (In Situ Cell Death Detection kit; Roche Molecular Biochemicals, Indianapolis, IN, USA). The TUNEL assay labels the characteristic DNA strand breaks of apoptosis with fluorescent nucleotides. Because normal cells have few strand breaks, little or no fluorescence is incorporated into normal cells. Nucleotides were labeled with fluorescein, which fluoresces green. Negative controls were performed with the TUNEL In Situ Cell Death Detection kit without applying the terminal transferase enzyme. Positive controls were performed using the TUNEL enzyme DNase I (30,000 U/mL) prior to the labeling procedure.

**Fig. 1:**
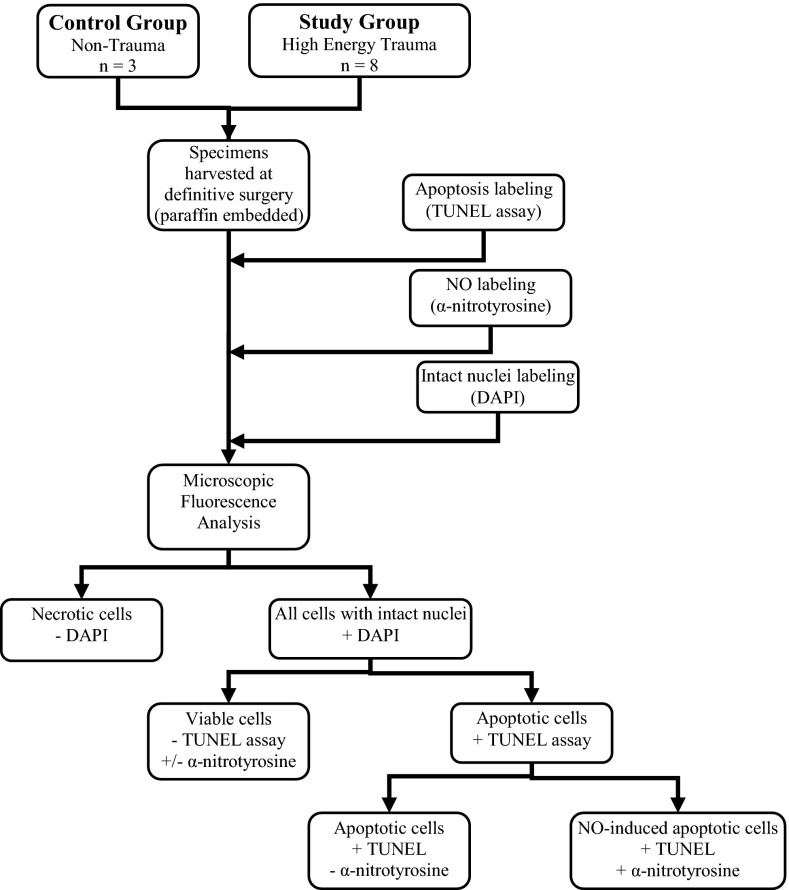
Study Design. Specimens from both groups were stained for all three assays and analyzed under fluorescence microscopy. Cells with intact nuclei were then determined to be apoptotic based on the TUNEL assay. Apoptotic cells were then sub-classified based on the presence of NO

**Fig. 2:**
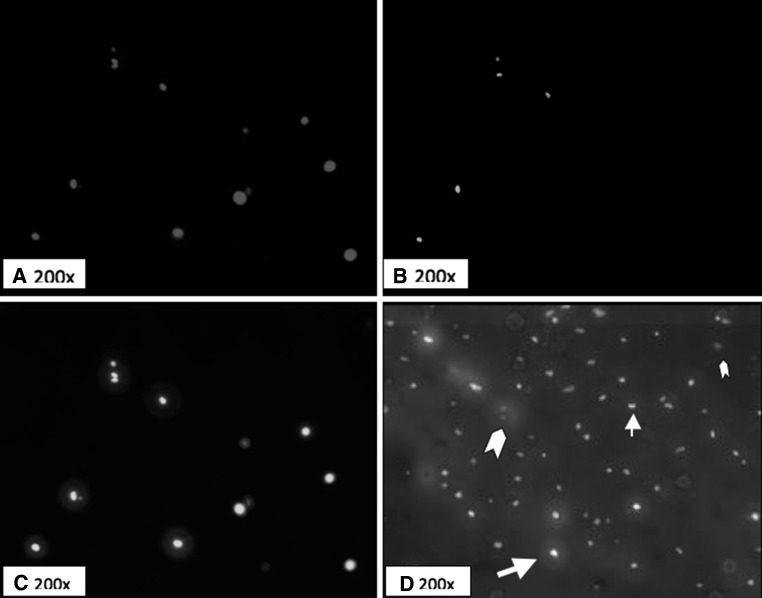
Fluorescence staining of cartilage slides. **a** Cells with intact nuclei (DAPI, ×200). **b** NO-containing cells (Rhodamine-X, ×200). **c** Apoptotic cells (TUNEL, ×200). **d** Computer-generated combined overlay image (×200). *Large arrow* indicates a cell staining positive for both NO and apoptosis. *Small arrow* indicates a cell staining positive for apoptosis and negative for NO. *Large arrowhead* indicates a cell positive for NO and negative for apoptosis. *Small arrowhead* indicates a cell negative for both NO and apoptosis

NO reacts with the tyrosine residues of intracellular proteins to form nitrotyrosine [15, 22]. Antibodies to nitrotyrosine can be used to mark sites of NO production [14, 23]. After staining for apoptosis with the TUNEL kit, the same tissue sections were blocked with 5 % normal goat serum and were then exposed to 1:200 anti-nitrotyrosine rabbit antibody (Sigma-Aldrich). We counterstained the sections with a 1:100 goat anti-rabbit antibody labeled with Rhodamine Red-X (Jackson ImmunoResearch, West Grove, PA, USA), which fluoresces red. Slides were then mounted in media containing 1.5 ug/mL 4′,6-diamidino-2-phenylindole (Vector Shield with DAPI; Vector Laboratories, Burlingame, CA, USA), a fluorescent blue nonspecific nuclear stain. Following the protocol, all cells with intact nuclei fluoresce blue, apoptotic cells fluoresce green, and cells containing NO fluoresce red under fluorescent microscopy (Figs. 1, 2).

We prepared three nonconsecutive slides from each cartilage sample. One image from each slide was photographed with a fluorescent microscope (Axiovert 200; Carl Zeiss Light Microscopy, Oberkochen, Germany) at 200× magnification under blue, green, and red fluorescence. The image was taken from the interior of the cartilage (to avoid the edge necrosis effect) in a nonrandom manner to capture a large number of cells in the frame. We only included cells with intact nuclei in the analysis. This resulted in a mean of 19 cells analyzed per section (range 7–45). The section was then photographed with blue, green, and red fluorescence, and the number of cells positive for each was counted (Fig. 2).

A total of 24 slides from the eight test specimens and 9 slides from the three control patients were analyzed. One of the authors (DEP) was blinded to the origin of the samples and reviewed each of the 33 slides. The observed variability by the reviewer between the 3 slides of each specimen was found to be 0.684. The percentage of cells staining positive for apoptosis and NO in each section was calculated based on the number of viable cells on each section. The three slides were then averaged to determine the value per specimen. In this manner, each specimen was weighted equally and cell density was thus normalized for each specimen. The percentage of cells positive for apoptosis and/or NO was calculated for the test and control specimens.

The percentage of cells that stained positive for both apoptosis and NO were compared with the percentage of cells staining positive only for apoptosis in the test specimens using Student’s *t* test, as the cells were all exposed to the same staining procedure and the means and variance for both populations were assumed to be equal. For each specimen, the percentage of cells with apoptosis and NO was plotted against time from injury and correlation coefficients were calculated (SPSS 15.0 for Windows; IBM, Somers, NY, USA).

## Results

There was no difference (*p* = 0.127) between the mean age of the trauma patients (47.8 years; range 23–56) and the control group (36 years; range 31–39). There was no difference in gender or comorbidities between the groups—there were 6 males in the trauma group and 1 male in the control group (*p* = 0.219) and 3 comorbidities in the trauma group and none in the control group (*p* = 0.148). The comorbidities of the trauma patients were hypertension, gastroesophageal reflux disease and anxiety, which the authors do not believe influenced the control specimens. The specimens were harvested at a mean time of 211 h (range 72–456) after injury and ranged in size from 4−16 mm in maximum dimension.

No difference in the percentage of intact cells, NO-positive cells, or apoptotic cells was found amongst the different anatomic locations—tibial plateau, tibial pilon, acetabulum, and calcaneus (*p* > 0.05). No difference was found in all outcomes between specimens harvested from the most frequent location, the tibial plateau and all other locations (*p* > 0.05). Additionally, no trend was found in the percentage of NO-positive cells or apoptotic cells based on the number of viable cells in each specimen (*p* > 0.05).

The percentage of cells with apoptosis was greater (*p* < 0.001) in the fracture group than in the control group—55.8 versus 4.3 %, respectively (Table 1). Similarly, the percentage of cells with NO was greater (*p* < 0.02) in the fracture group than in the control group—39.2 versus 10.9 %, respectively (Table 1).

**Table 1:** Percentage of NO and apoptosis in fracture and control cartilage samples

Percentage of chondrocyte staining positive	Fracture cartilage		Control cartilage		*p*
	*n* = 24 (range)	SD (%)	*n* = 9 (range)	SD (%)	
Apoptosis (TUNEL)	55.8 % (6–100)	30.9	4.3 % (0–15)	5.7	<0.001
NO (α-nitrotyrosine)	39.2 % (6–100)	31.7	10.9 % (0–31)	12.2	<0.02

*n* is the number of microscopic slides reviewed; percentage was calculated per section of cartilage

For chondrocytes staining positive for apoptosis, the percentage of cells co-staining for NO was greater (*p* < 0.001) in the fracture group than in the control group—58.7 versus 19.6 % (Table 2). There was no difference in percentage of cells co-staining for NO and apoptosis in the control subjects. Given the small sample size, a sensitivity analysis for outliers was performed and found no individual samples had a significant skew effect on the data to be considered an outlier.

**Table 2:** Prevalence of nitric oxide in apoptotic chondrocytes in fracture

Positive for nitric oxide	Positive for apoptosis	SD	Negative for apoptosis	SD	P
Fracture group *n* = 24	58.7 % (0–100)	36.3 %	19.6 % (0–93)	28.7 %	<0.001
Control group *n* = 9	11.1 % (0–100)	33.3 %	9.9 % (0–27)	10.4 %	0.919

Percentage was calculated per section of cartilage; *n* is the number of chondrocytes evaluated in each group

**Fig. 3:**
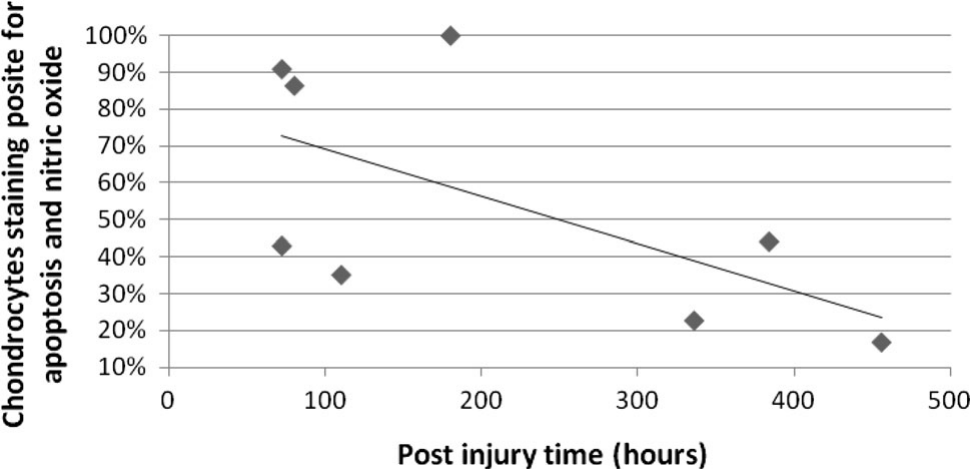
Timing of chondrocyte apoptosis. The rate of chondrocyte apoptosis has a moderate correlation of 0.62 with a coefficient of demonstration (*r* ^2^) of 0.38 (*p* = 0.01) indicating that 38 % of the decreasing rate of chondrocyte apoptosis is the result of increasing time from injury

There was a negative correlation of −0.615 (*p* = 0.01) between the percentage of chondrocytes positively co-staining for apoptosis and NO and increasing time from injury (Fig. 3); the coefficient of determination for this correlation was 38 %. There was no correlation between the percentage of cells staining positive independently for NO, apoptosis, or the total number of intact cells with time from injury. There were insufficient data points to calculate a best-fit trend line to determine if the rate of apoptosis was logarithmic, linear, or variable.

## Discussion

Chondrocyte apoptosis has been identified in osteoarthritic cartilage in animal models and in human samples taken at the time of implant arthroplasty [12, 14]; however, more recently it has also been implicated in post-traumatic osteoarthritis [39–41]. Cyclic loading [24], matrix lacerations [25], and blunt impact [8, 26–28] all decrease chondrocyte viability in cartilage explants. It is speculated that loss of chondrocytes is a key step in the development of arthritis; without cells to maintain the cartilage matrix, the joint inevitably progresses to osteoarthritis. Studies have also identified NO in arthritic cartilage [12, 14], while others found NO could induce apoptosis in cultured chondrocytes [12]. NO synthase inhibitors seemed to reduce the progression of arthritis in animal models [29, 30], suggesting NO may be a signal for human chondrocyte apoptosis in vivo [18]. Chondrocytes subjected to high-energy trauma undergo apoptosis [16]. This first aim of this study was to identify the presence of NO-induced apoptosis in traumatized human chondrocytes after injury. The second aim of this study was to attempt to demonstrate a greater degree of NO-induced apoptosis in chondrocytes subjected to high-energy trauma compared to non-traumatized chondrocytes, indicating that the apoptosis in these chondrocytes is due to the presence of NO. The study also attempted to identify the timeline of chondrocyte NO-induced apoptosis after high-energy injury.

We acknowledge limitations to our study. First, we had a small number of specimens from various joints, with varying forces and orientations across each cartilaginous surface. It is unclear how these differences influence the rate, induction, or timing of chondrocyte apoptosis; however, no significant differences were found in all outcomes between the specimens harvested from the various locations, implying that NO-induced apoptosis is not influenced by the location or force of the fracture, but may be a unified down-stream result of intra-articular damage. Despite, the small size, a power analysis using the eight study subjects and three control subjects yielded a power of 0.91 to determine differences in the percentage of cells staining positive for NO and apoptosis between the control and fracture groups. This was based on a determined standard deviation of 30.3 % and a 0.05 two-tailed significance level [31]. Second, co-localization of NO and apoptosis does not imply causality, merely correlation; however, other studies have elucidated the role of NO in apoptosis [17, 22, 32–34]. The study design did not allow for determination of the intra- and inter-observer variability of the methods; however, the variability between the three specimens from each specimen was determined to be good (*R* = 0.684). Third, the specimens used were small pieces that would otherwise be discarded at the time of surgery. It is likely that these samples are the most traumatized sections of the cartilage, resulting in a sampling error that biases the results toward the null hypothesis and may not be representative of the remainder of the articular cartilage; however, these fragments may be more subject to mechanical cell death rather than apoptotic cell death subsequently, which would bias the results against the hypothesis of this study. Fourth, the controls were not normal cartilage because they was harvested from midfoot joints with deformity and pain and the properties of the cartilage of this joint may be different from traumatized joints. This bias would favor the null hypothesis showing no difference with traumatized cartilage regardless of the underlying cause. It is possible that using arthritic cartilage as a control would only lead to a bias towards rejecting the hypotheses of this study, because the control cartilage is more likely to have higher rates of cell death than completely normal cartilage. Fifth, the TUNEL assay was the primary test for apoptosis in this study, but the TUNEL assay has been criticized for accurately detecting apoptosis [35]. Other studies have compared the TUNEL assay to other methods of detection such as enzyme-linked immunosorbent assay (ELISA), flow cytometry, and caspase-3 assays, finding good correlation among the various methods [32–34]. Although the TUNEL assay and nitrotyrosine stains were titrated carefully in control and fracture samples so as not to give false-negatives or false-positives, these techniques are inherently subject to both positive and negative error. The data should be interpreted more qualitatively than quantitatively.

This study found a higher percentage of chondrocytes staining independently for apoptosis in fracture specimens than in the control specimens. This concurs with Murray et al. [16] who found a high percentage of apoptotic cells in cartilage samples from patients with intra-articular fractures. Sena et al. [41] found similar results in cultured chondrocytes from calcaneal fractures that had sustained high-energy trauma.

Second, our study found a greater percentage of traumatized chondrocytes showed co-staining for NO and apoptosis compared to chondrocytes from control specimens. This supports the role of NO-induced apoptosis after high-energy trauma in human chondrocytes. Blanco et al. [17] hypothesized, based on the study of cultured chondrocytes, that NO is the primary inducer of apoptosis. Although NO is not the sole catalyst of post-traumatic chondrocyte death, it plays a central role [15, 35, 36]. Several studies suggest several factors inhibit the NO-induced apoptotic pathway, including hyaluronic acid, cilostazol, and other agents [15, 17, 30, 33–38]. A better understanding of the chondrocyte apoptotic pathway could lead to intervention and prevention of chondrocyte death. Administration of NO-inhibiting agents at the time of initial presentation or surgery may be a novel treatment in the future [15].

Finally, our study found persistent co-staining of NO and apoptosis with increasing time from injury as a negative correlation, which is supported by the literature. Lima et al. [26] found chondrocyte death increased until 7 days after a single impact injury, and the percentage of dead cells was decreased by apoptosis inhibitors. In a bovine cartilage explants model, Loening et al. [32] showed via TUNEL staining that apoptosis peaked 24 h after the simulated loading event and persisted for 2 days and other signs of cartilage injury increased for 6 days. Tew et al. [9] also found in bovine cartilage explants damaged by cutting that apoptotic cells detected by TUNEL staining remained in the tissue for 20 days following injury. This study, with few time points cannot determine the rate or the pattern of NO-induced apoptosis to be parabolic, logarithm, linear or variable. Additional time points would aide in affirming the theory that apoptosis increases initially and diminishes with increasing time to injury. More detailed studies analyzing the timing of the NO cascade in vivo are necessary to delineate this pathway.

In conclusion, we found the percentage of cells with apoptosis is increased in cartilage from trauma patients, and that those cells are more likely to contain NO, implicating NO-induced apoptosis after intra-articular fracture. Our study found a decreasing number of NO-induced apoptotic chondrocytes with increasing time from injury without being able to show the specific time course of apoptosis after trauma. Future studies should focus on animal models to assess the rate of chondrocyte cell death due to NO-induced apoptosis compared to other causes of both immediate and delayed cell death as well as determining the viability of remaining chondrocytes to survive. The degree to which NO-induced apoptosis is the ultimate cause of post-traumatic arthritis will have to be further elucidated with models both inducing and blocking NO-apoptosis in animal models. This should be performed in both normal and traumatized cartilage to account for the presence and contribution of post-traumatic fibrocartilage after injury. Additional time points will be necessary to elucidate the rate and pattern of NO-induced apoptosis. We believe it is important to focus on NO-induced apoptosis because of existing therapies to block NO that may be applicable to patients undergoing intra-articular fractures.
